# Soluble Solids Content Binary Classification of Miyagawa Satsuma in Chongming Island Based on Near Infrared Spectroscopy

**DOI:** 10.3389/fpls.2022.841452

**Published:** 2022-07-18

**Authors:** Yuzhen Chen, Wanxia Sun, Songtao Jiu, Lei Wang, Bohan Deng, Zili Chen, Fei Jiang, Menghan Hu, Caixi Zhang

**Affiliations:** ^1^School of Agriculture and Biology, Shanghai Jiao Tong University, Shanghai, China; ^2^Shanghai Key Laboratory of Multidimensional Information Processing, School of Communication and Electronic Engineering, East China Normal University, Shanghai, China; ^3^Shanghai Citrus Research Institute, Shanghai, China

**Keywords:** near infrared spectroscopy, AdaBoost, random frog, citrus soluble solids content, machine learning

## Abstract

Citrus is one of the most important fruits in China. Miyagawa Satsuma, one kind of citrus, is a nutritious agricultural product with regional characteristics of Chongming Island. Near-infrared Spectroscopy (NIR) is a proper method for studying the quality of fruits, because it is low-cost, efficient, non-destructive, and repeatable. Therefore, the NIR technique is used to detect citrus's soluble solid content (SSC) in this study. After obtaining the original spectral data, the first 70% of them are divided into the training set and 30% into the test set. Then, the Random Frog algorithm is chosen to select characteristic wavelengths, which reduces the dimension of the data and the complexity of the model, and accordingly makes the generalization of the classification model better. After comparing the performance of various classifiers (AdaBoost, KNN, LS-SVM, and Bayes) under different characteristic wavelength numbers, the AdaBoost classifier outperforms using 275 characteristic wavelengths for modeling eventually. The accuracy, precision, recall, and *F*_1_-score are 78.3%, 80.5%, 78.3%, and 0.780, respectively and the ROC (Receiver Operating Characteristic Curve, ROC curve) is close to the upper left corner, suggesting that the classification model is acceptable. The results demonstrate that it is feasible to use the NIR technique to estimate whether the citrus is sweet or not. Furthermore, it is beneficial for us to apply the obtained models for identifying the quality of citrus correctly. For fruit traders, the model helps them to determine the growth cycle of citrus more scientifically, improve the level of citrus cultivation and management and the final fruit quality, and thus increase the economic income of fruit traders.

## 1. Introduction

Citrus fruits are among the most commonly grown and consumed fruits all over the world and meanwhile one of the most important fruits in China since they are very nutritious and can supplement vitamins, promote digestion and increase appetite (Zou et al., 2016; Anticona et al., 2020). The total output of *Citrus reticulata Blanco* is 21.2 million tons in China, accounting for 67% of the total citrus output. *Citrus unshiu* is one of the three main varieties of citrus reticulata Blanco in China (Nam et al., 2019; Cheng et al., 2020). This research uses Miyagawa Satsuma, a variety of *citrus unshiu*, from Chongming Island in Shanghai, as the research object. The citrus in Chongming Island not only grows in environmental conditions famous for fresh air, clean water, and rich soil but also ripens in cultivation technology of “green prevention and control and plastic film covering with grass and organic cultivation” (this cultivation concept originates from Shanghai Qianwei Citrus Co., Ltd.). As a result, it owns the advantages of both rich nutrition and the regional characteristics of Chongming Island.

Soluble solids content (SSC), is one of the most important internal quality attributes of most fruits. The SSC plays an important role in the fruit maturity process and partly influences the flavor of most fruits, thus determining the acceptance of rich nutrients and economic benefits in the fruit trade. The detection of citrus SSC is not only beneficial to customers but also significant for growers (Li et al., 2016b; Fan et al., 2019; Guo et al., 2020). Therefore, in recent decades, the demand to develop non-destructive and rapid evaluation methods for citrus SSC has become more extensive and urgent. Electronic nose technology (Zhang et al., 2008, 2016), computer vision (Xia et al., 2016; Bhargava and Bansal, 2021), and hyperspectral imaging technology (Li et al., 2016a, 2018) are some common methods to measure the quality of fruits. However, electronic nose technology is restricted to limited enclosed space, which is inconvenient to carry out. Computer vision technology lacks spectral information. As for hyperspectral imaging technology, the obtained hypercube contains a lot of redundant information which leads to a high computation cost.

Fortunately, with the advantage of low testing cost, high efficiency, good reproducibility of test results, and non-destructive testing, NIR spectroscopy, between wavelength region range of 780–2,526 nm, has been applied popularly in the analysis of different fruit or vegetable samples (Beghi et al., 2017; Arendse et al., 2018), such as apple (Xia et al., 2020; Arefi et al., 2021; Ma et al., 2021; Li et al., 2022), tomato (Huang et al., 2021; Zhang et al., 2021), persimmon (Wei et al., 2020), pear (Cruz et al., 2021), and banana (Cruz et al., 2021). Xia et al. (2020) studied the effect of sample diameter differences on the online prediction of SSC of “Fuji” apples with the methods of visible and near-infrared spectroscopy and partial least square regression. It is justified that diffuse transmission spectra in 710–980 nm and diameter correction method with calculated attenuation coefficient are the best. Wei et al. used NIR hyperspectral imaging within 900–1,700 nm to model SSC and firmness determination of persimmon with partial least squares regression (Wei et al., 2020). The final models obtained a coefficient of determination of 0.757, RMSEP of 1.404 Brix, and $R_{p}^{2}$ of 0.876, RMSEP of 0.395 for SSC and firmness detection, respectively. Pahlawan et al. developed the calibration model to predict the SSC of bananas using NIR spectroscopy in the range from 350 to 1,000 nm. It was conducted by various distances of fiber optic probes to bananas samples (Cruz et al., 2021). To our best knowledge, there have been few similar studies on Miyagawa Satsuma. From these researches mentioned above, it can be easily seen that they focus on predicting the accurate number of the attribute focused on, such as SSC. Sometimes, we are more interested in knowing the sugar level rating rather than the specific value. There is no exact numerical index for distinguishing sweet and unsweetened, and therefore, we calculate the average value of SSC as the demarcation index for judging sweet or unsweet for the reason that SSC is an important index affecting the sweetness.

Reducing dimensions and seeking the most informative wavelengths are effective methods for processing data while selecting the most informative wavelengths of target information is an effective measure to simplify computation and improve the model performance (Li et al., 2019; Zhou et al., 2020). First, it has been shown that the inclusion of uninformative wavelengths while modeling affects the performance of predicting or classifying and model interpretability (Chang et al., 2016). Second, the identification of wavelengths that contain information about the attribute the research focuses on, will reduce the computation time and cost, from a more practical point of view (Zhang et al., 2019; Mamouei et al., 2020). Li et al. (2016a) chose the carlo-uninformative variable elimination and successive projections algorithm to select the most effective variables from hyperspectral data when doing the research on measuring SSC in pear. The results indicated that the model built using 18 effective variables achieved the optimal performance for the prediction of SSC. Jun et al. (2018) used an iteratively retaining informative variables algorithm to obtain 10 characteristic wavelengths when processing samples in predicting the SSC of cherry tomatoes. The experimental results showed the IRIV−CS−SVR model for SSC prediction could reach accuracy with $R_{p}^{2}$ = 0.9718 and $R_{c}^{2}$ = 0.9845. Fan et al. (2014) adopted a combination of the standard normal variate, uninformative variable elimination, genetic algorithm, and successive projections algorithm to obtain 30 characteristic wavelengths selected from full-spectra achieving the optimal performance.

In the current study, the binary classification of Miyagawa Satsuma is focused on, which owns the regional characteristics of Chongming Island. The classification model for nondestructive determination of Miyagawa Satsuma SSC will judge the quality of citrus more scientifically, and overcome the shortcomings of subjective differences and low efficiency. Meanwhile, it can identify the growth cycle of citrus and estimate the maturity time more accurately, which is conducive to the management arrangement such as picking. It can also provide a theoretical basis for citrus grading, which is good for fruit farmers or manufacturers to sell graded citrus and improve profits (Kundu et al., 2021).

## 2. Materials and Methods

### 2.1. Data Collection

#### 2.1.1. Near Infrared Spectra Acquisition

The equipment employed in our research is the Fourier transform near infrared spectrometer, an antaris II–F-NIR analyzer made by Thermo Fisher. We set NIR acquisition mode as integrating sphere mode and the gain as ×1. The NIR spectra are within the range from 1,000 nm to 2,500 nm.

**Figure 1:**
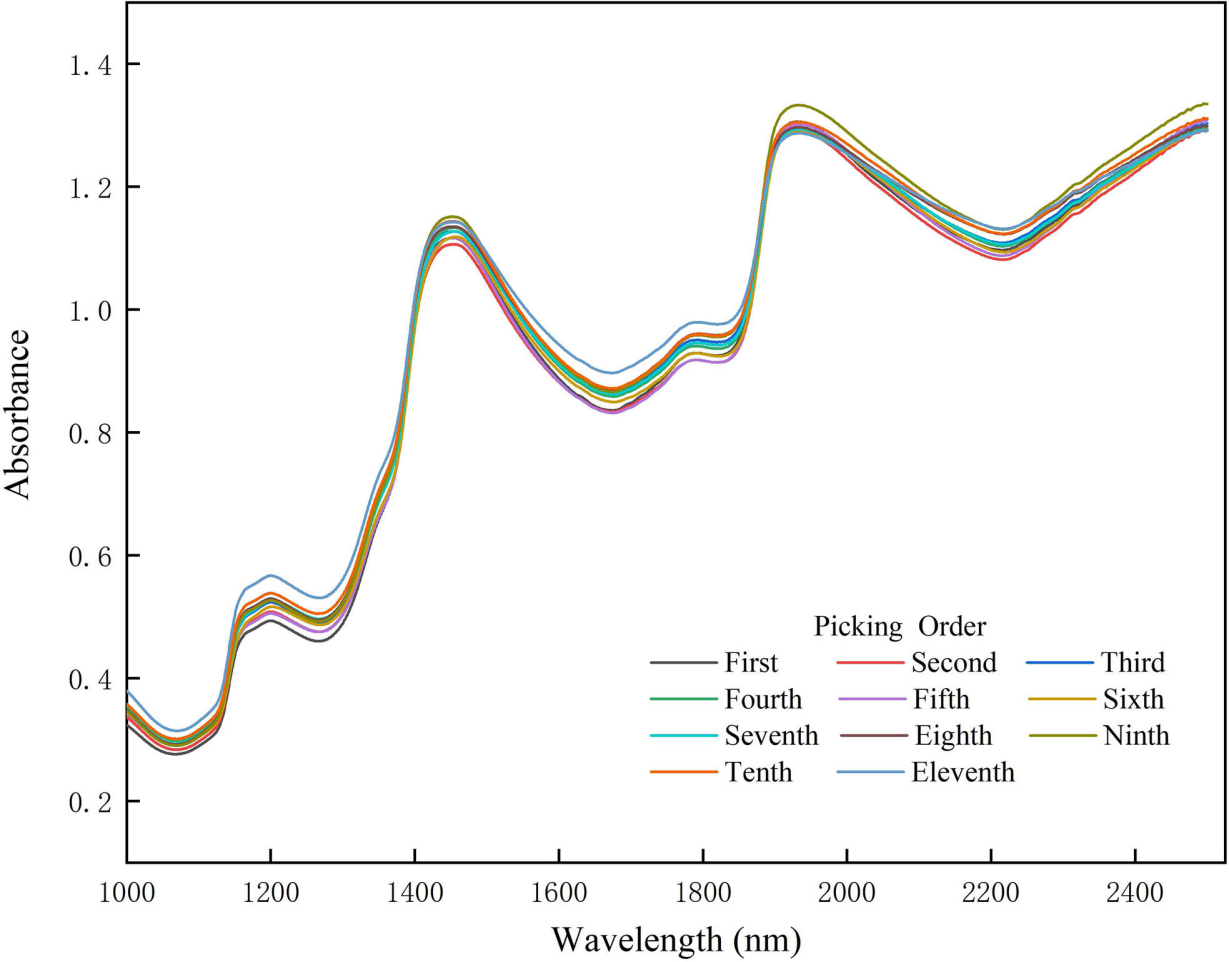
Near infrared (NIR) spectra of citrus in different picking times in chronological order. Different picking orders are represented by different colors. Each line is the average spectrum of 12 fruit samples at each picking time.

All samples of Miyagawa Satsuma came from Shanghai Qianwei citrus Co., Ltd located on Chongming Island. All samplings, 11 times total, were carried out within 3 months. In each sampling, five trees with the most similar growth were selected, which were without films and not among the outermost three rows of trees. Then the five trees were divided into upper, middle, and lower parts, where one sample was picked, respectively, from four directions: south, east, north, and west. As a result, 12 samples were obtained per tree, and a total of 60 were taken for each sampling. Next, 12 samples were randomly chosen among a total of 60 fruits. For each sample in the 12 fruits, the NIR spectra were gained from 4 points at the cross symmetry of the equatorial plane of the fruit. Finally, the averaged NIR spectra, obtained by averaging NIR spectra of four points, were taken as the original NIR spectra, as shown in Figure 1.

#### 2.1.2. Soluble Solids Content Acquisition

After the Miyagawa Satsuma was squeezed and centrifuged, the SSC of the selected Miyagawa Satsuma samples was measured with a saccharometer (PR-101; Atago Co., Tokyo, Japan).

### 2.2. Data Preprocessing

The samples with obviously incomplete or wrong data are eliminated, whether NIR spectra or SSC, thus obtaining a total of 122 samples. Then samples were divided into the training set and test set by the SPXY algorithm (Galvao et al., 2005), with 70% of the samples as the training set and 30% as the test set. The principle of the Kennard stone algorithm (KS) algorithm is to calculate the Euclidean distance among all samples: select two samples with the maximum Euclidean distance into the training set, then carry out the iterative calculation, select the samples with the maximum and minimum Euclidean distance into the training set until the number of samples required by the training set is reached. SPXY algorithm is based on the KS algorithm, and it furthermore involves the chemical values and spectra among samples when calculating Euclidean distance, which makes the training set more representative, and makes the generalization ability of the established prediction model better.

### 2.3. Characteristic Wavelength Selection

The random frog (RF) algorithm (Li et al., 2012) was used to obtain the corresponding number of characteristic wavelengths of NIR spectral data, which has the features of conceptually simplicity, and fewer parameters to be trained in algorithm implementation, strong global search and optimization ability, etc. The principles of the algorithm are as follows. Each sample in a population is regarded as a frog. Then the whole population is divided into *m* sub-groups with the scale of *n*. In each sub-group, the frogs with the best and worst fitness are used to produce a new child frog, which can be viewed as a jump of the best frog. If the fitness of the child frog is better than the parent with the worst fitness, replace the worst parent with the child, otherwise, randomly generate a new child, which can be viewed as the best frog's jumping again. If the fitness of the new child frog is still worse than the worst parent, then randomly generate another new child to replace the parent with the worst fitness. The evolutionary strategy of the random frog algorithm is like frogs jumping toward the optimal solution so that the algorithm gradually converges to the optimal solution.

The more specific steps of this algorithm are as follows: First, initialize parameters. Second, randomly generate an initial frog group and calculate the fitness of each frog. Third, arrange the frogs in descending order according to the value of fitness, and record the local optimal solution *P*_*x*_. Then divide the *F* frogs from the initial group into sub-groups, namely, allocate *F* frogs into *m* sub-groups with the scale of *n*. Fourth, do a local search process, i.e., do the process described above in each sub-group. As a result, sub-groups do the fourth process, redivide the frog group, do the same operation as the first round, and record the global optimal solution *P*_*x*_. Fifth, verify the calculation stop condition. If the convergence conditions of the algorithm are reached, the RF algorithm ends. If the global optimal solution has not been significantly improved, the execution of the algorithm should also be stopped.

To validate the performance of the RF algorithm in this task, the other common wavelength selection namely the competitive adaptive reweighted sampling algorithm (CARS) is used for comparison with the RF algorithm.

### 2.4. Binary Classification Model

The AdaBoost classifier (Freund et al., 1999) is selected for modeling. Boosting is an important integrated learning technology, which can enhance weak classifiers with poor prediction performance into strong classifications with good prediction performance in a cascade way. The core of its adaptability is that the wrong samples of the previous basic classifier will be strengthened, and all the weighted samples will be used to train the next basic classifier again. At the same time, a new weak classifier is added in each round until a predetermined small enough error rate or a predetermined maximum number of iterations is reached.

Specifically, the entire AdaBoost iterative algorithm consists of three steps: First, initialize the weight distribution of training data. If there are *n* samples, each training sample is given the same weight of 1/*n* at the beginning. Second, train weak classifiers. In the specific training process, if a sample point has been accurately classified, its weight will be reduced in the construction of the next training set; On the contrary, if a sample point is not accurately classified, its weight will be improved. Then, the weight updated sample set is used to train the next classifier, and the whole training process goes on in this way, iteratively. Third, combine the trained weak classifiers into strong classifiers. After the training process of each weak classifier, increase the weight of the weak classifier with a small classification error rate to make it play a greater decisive role in the final classification function while doing the opposite operation for the weak classifier with a large classification error rate.

To compare the performance of various classifiers, we choose AdaBoost, k-Nearest Neighbor (KNN), Bayes classifier, and LS-SVM to explore the best-performing classification model. In the current study, we use Matlab and Weka to establish the models.

### 2.5. Model Evaluation

To verify the efficiency of the classification system, evaluation indicators viz. confusion matrix, accuracy, precision, recall, *F*_1_, micro-measures, and macro-measures are considered.

1) Confusion matrix: Assume that “Positive” means the positive samples and that “Negative” means the negative samples. Meanwhile, “True” represents that the prediction is right while “False” represents that the prediction is wrong. As a result, “TP” and “TN” mean that the positive sample is classified as “Positive” and that the negative sample is labeled as “Negative”, respectively. “FP” and “FN” represent that the negative sample is labeled as “Positive” and that the positive sample is classified as “False.” The four indicators make up the confusion matrix.

2) Accuracy: It is a ratio that is used to estimate the classification ability of a model within the range from 0 to 1. Generally speaking, the larger accuracy is, the better the classification is. It can be calculated by the following equation:


(1) $$\begin{array}{l} Accuracy=\frac{TN+TP}{TN+TP+FP+FN} \end{array}$$

3) Precision: Precision is only used to evaluate the classification ability of the positive samples within the range from 0 to 1. It is obvious that the larger precision is, the more effective the system is. It is computed by:


(2) $$\begin{array}{l} Precision=\frac{TP}{TP+FP} \end{array}$$

4) Recall: It is a ratio from 0 to 1. Obviously, the more it is close to 1, the better the system is. The calculation equation is:


(3) $$\begin{array}{l} Recall=\frac{TP}{TP+FN} \end{array}$$

5) *F*_1_: It is a harmonic mean of recall and precision. In this study, we consider the weight of recall and precision the same, which means attaching the weight of 0.5 to either of them. It is calculated by:


(4) $$\begin{array}{l} F_{1}=\frac{2*Precision*Recall}{Precision+Recall} \end{array}$$

6) Receiver Operating Characteristic (ROC) Curve and Area Under Curve (AUC): The abscissa of the ROC curve is the false positive rate (FPR) while the ordinate is the true positive rate (TPR), where $\text{FPR}=\frac{FP}{TN+FP}$ and $\text{TPR}=\frac{YP}{TN+FN}$. Generally speaking, the closer the ROC curve is to the upper left corner of the image, the better the performance of the binary classifier. AUC is the area under the ROC curve and it is generally within the range of (0.5, 1). When the closer the ROC curve is to the upper left corner, the greater the value of AUC.

## 3. Experimental Results and Analysis

### 3.1. NIR Spectral Characteristics of Miyagawa Satsuma in Different Picking Time

The NIR spectra in different picking times are shown in Figure 1. The trend of the citrus spectra collected each time is similar. There is an obvious absorption trough near 1,080, 1,300, 1,700, and 2,200 nm, respectively. According to the principles of NIR spectroscopy, due to the fact that the sample will selectively absorb NIR waves with different frequencies, the NIR wave which passes through the sample will become weaker in some wavelength ranges, and the transmitted NIR wave will carry the information of organic component and structure. Therefore, it can be inferred that these absorption troughs can probably be the most informative areas, which can be reflected in the characteristic wavelength selection.

Our reason for using fruits with different picking periods for modeling is to increase the coverage of the SSC, allowing a larger range of variation in the spectral data and ultimately increasing the model robustness. We performed the statistical tests on the obtained spectral data and SSC and found significant differences between spectral data and SSC for non-adjacent picking periods (*p* < 0.05) and no significant differences for adjacent picking periods (*p* > 0.05). This is in accordance with expectations. Because, as the fruit ripens, the SSC will certainly increase and the spectral differences will increase.

### 3.2. Performance of RF

**Figure 2:**
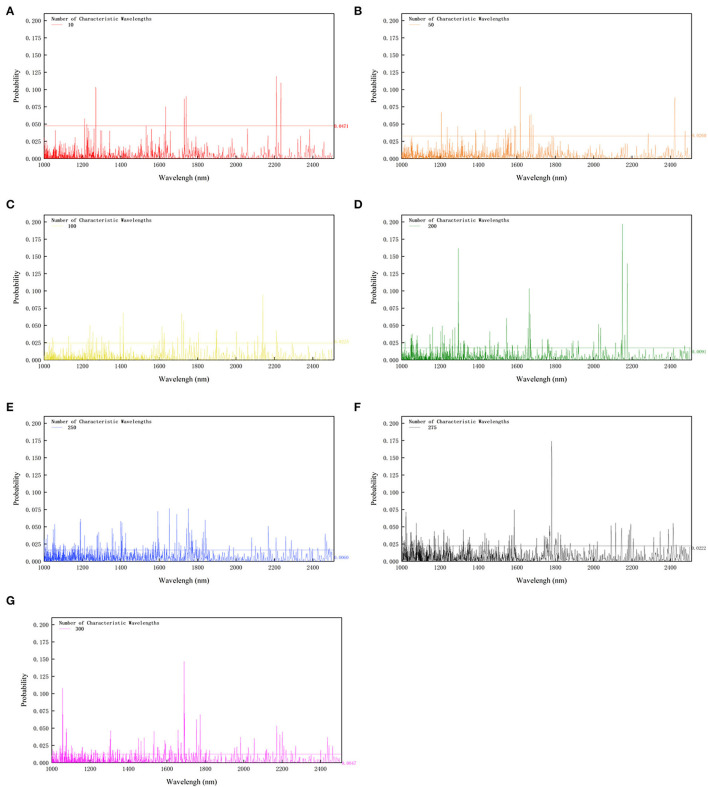
Cutoff probability of different characteristic wavelengths. For the subsequent modeling, 10 **(A)**, 50 **(B)**, 100 **(C)**, 200 **(D)**, 250 **(E)**, 275 **(F)**, and 300 **(G)** characteristic wavelengths are chosen, respectively. The numbers on the right corner of the figure are the cutoff probability of corresponding characteristic wavelengths number. The cutoff probability generally decreases as the number of informative wavelengths increases.

As mentioned above, the characteristic wavelength selection can accelerate the computation speed and reduce computation cost to a degree. RF algorithm is chosen to generate characteristic wavelengths with the numbers 10, 50, 100, 200, 250, 275, and 300, respectively, which is displayed in Figure 2. It is easy to find that the larger the number of characteristic wavelengths is, the smaller the cutoff probability is. The cutoff probability indicates the threshold value for screening the required number of informative wavelengths. The wavelength numbers, 10, 50, 100, 200, 250, 275, and 300, respectively correspond to the cutoff probabilities, 0.0471, 0.0260, 0.0225, 0.0222, 0.0091, 0.0060, and 0.0047. The cutoff probability generally decreases as the number of informative wavelengths increases (Figure 2). Meanwhile, it is true with what has been inferred in the above section that the absorption troughs can be the most informative, most of the retaining wavelengths gather in the areas inferred before viz. 1,080, 1,300, 1,700, and 2,200 nm. This probably has a relationship with the functional groups viz. —OH, —CH, —NH.

In addition, the classification models based on CARS selected wavelengths are established, and their performance is not as good as the RF-based models. For example, when ten characteristic wavelengths are selected, the RF-based model gives a better performance than the model based on CARS, with the accuracy of 60.9, 69.6, 65.2, and 62.2% vs. 52.78, 52.78, 58.33, and 47.22% for AdaBoost, KNN, Bayes, and LS-SVM modeling methods, respectively. Overall, CARS does not perform as well as RF for the informative wavelength selection.

### 3.3. Model Analysis Using Plant Physiology Phenomenon

The spectral properties of plants are mainly determined by their internal structure. For the current study, the obtained spectra are the result of the interaction of the incident light with the chemical composition and physical structure of Citrus. For Miyagawa Satsuma, its structure can be divided into exocarp (oil cell layer), mesocarp (white cortex), endocarp, fruit, and fruit stem from outside to inside. Among them, the surface of the soluble dietary fiber of mandarin pulp is not smooth, the strips and gaps are intertwined, and there are raised particles; the surface of the soluble dietary fiber of mandarin peel is larger, but the surface depressions are mixed with a few spherical particles. There is a strong interaction between the two molecules.

As an important indicator for evaluating fruit sweetness, SSC is mainly composed of soluble sugars (including sucrose, fructose, and glucose). In the NIR region, the stretching and deformation vibration absorption peaks of *O*−*H* bonds in soluble sugars are located around 1,440 and 2,080 nm, and there are three absorption peaks of soluble solids at 980, 1,169, and 1,485 nm (Musingarabwi et al., 2016). The water content has a great influence on the absorption of the plant spectrum. Under the condition of multi-layer leaves, the water absorption bands at 1,100 and 960 nm have a great influence on spectral reflectance. Absorption leads to a decrease in reflectance and an increase in absorbance, and peaks of reflectance (i.e., peaks and valleys of absorbance) appear at 1,600 and 2,200 nm (Ma et al., 2017).

The wavelengths selected by RF include three characteristic wavelengths near the absorption peaks of soluble solids at 980 nm, 1,169 nm, and 1,485 nm, and two characteristic wavelengths near the absorption peaks of stretching and deformation vibrations of *O*−*H* bonds in soluble sugars at 1,440 nm and 2,080 nm, and a characteristic wavelength near the strong absorption peak of water at 1,400 nm. This analysis explains why the model based on the RF selected wavelengths performs better.

### 3.4. Soluble Solids Content Division

**Figure 3:**
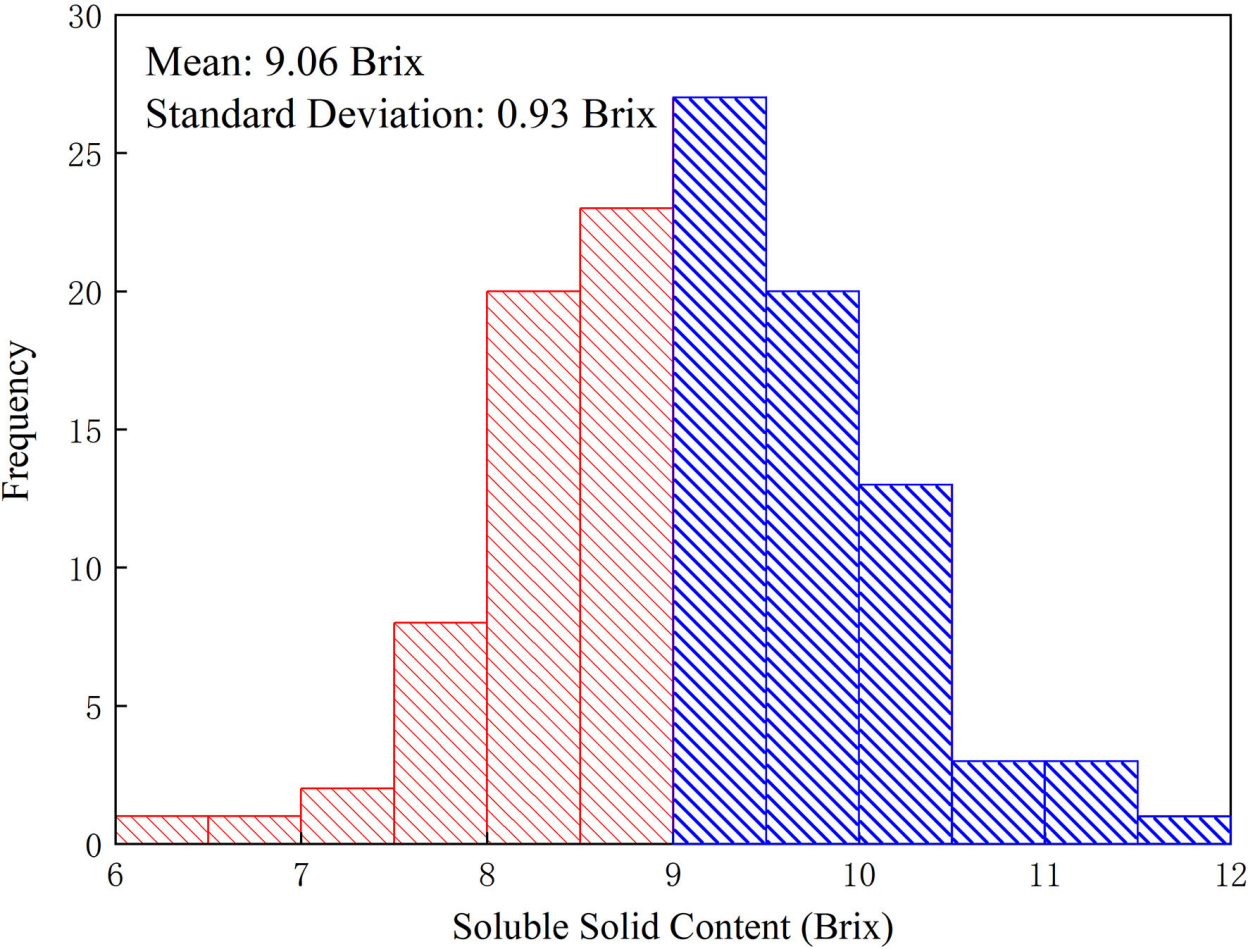
Histogram of citrus soluble solid content (SSC) of the total 122 fruit samples collected in this study. The mean of all citruses' SSC is 9.06 Brix while the SD is 0.93 Brix. The citruses corresponding to the blue areas are regarded as sweet while the citruses corresponding to the red ones are regarded as unsweet.

The research holds the opinion that consumers are more concerned about whether the Miyagawa Satsuma is sweet or not, but not the concrete value of sweetness. Referring to Figure 3, the dichotomous map or histogram of 122 Miyagawa Satsuma citruses' SSC, the distribution of this figure is roughly similar to the normal distribution, whose mean of all citruses' SSC is 9.06 Brix and the SD is 0.93 Brix. To carry out our belief, 9 Brix was taken as the boundary after asking an expert in agriculture for advice. As a consequence, the citruses with SSC more than or equal to 9 Brix are considered to be sweet and the others are not sweet for the following classification modeling.

**Figure 4:**
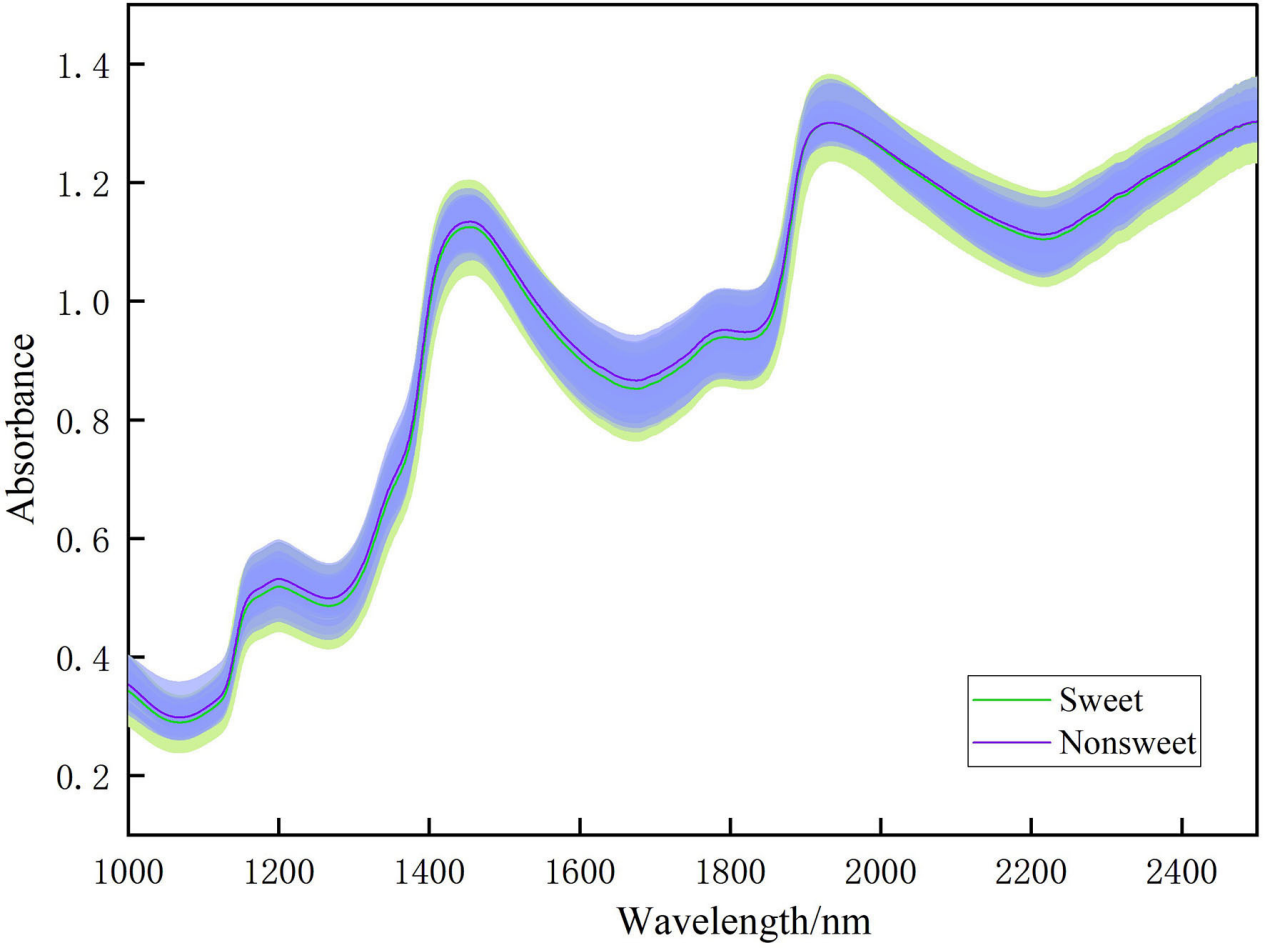
Comparison of NIR spectra of sweet (SSC beyond 9 Brix) and unsweet (SSC below 9 Brix) fruit samples. The shaded areas represent the confident intervals to each line.

Figure 4 shows the comparison of NIR spectra of sweet and unsweet fruit samples. As shown in Figure 4, the large overlap between the spectral curves of the sweet and unsweet samples indicates that the model will not perform as expected if the model is constructed based on original spectra. Therefore, we need to select the informative wavelengths specific to SSC classification, and then combine them with pattern recognition methods for modeling.

### 3.5. Performance Analysis of Different Binary Classification Models

As mentioned before, AdaBoost, KNN, Bayes, and LS-SVM are adopted to establish classification models. The performance comparison of different classifiers under different characteristic wavelengths is shown in Table 1. The conclusion can be drawn that when the number of characteristic wavelengths is 275, the classification model established by the AdaBoost classifier performs best (bold in the table), with accuracy, precision, recall, and *F*_1_-score 78.3%, 80.5%, 78.3%, 0.780, respectively.

**Table 1:** Modeling results of sweet (SSC beyond 9 Brix) and unsweet (SSC below 9 Brix) classification of Miyagawa Satsuma in Chongming Island under different classifiers with different characteristic wavelengths.

**Characteristic wavelengths**	**Models**		**Metrics**		
		**Accuracy**	**Precision**	**Recall**	**F1-score**
		**(%)**	**(%)**	**(%)**	
10	AdaBoost	60.9	62.1	60.9	0.604
	KNN	69.6	69.8	69.6	0.696
	Bayes	65.2	65.4	65.2	0.648
	LS-SVM	62.2	53.3	100.0	0.696
50	AdaBoost	60.9	62.1	60.9	0.604
	KNN	52.2	53.7	52.2	0.503
	Bayes	65.2	65.4	65.2	0.648
	LS-SVM	67.6	57.1	100.0	0.727
100	AdaBoost	69.6	69.8	69.6	0.696
	KNN	52.2	52.9	52.2	0.516
	Bayes	69.6	69.6	69.6	0.694
	LS-SVM	59.5	51.6	100.0	0.681
200	AdaBoost	65.2	71.6	65.2	0.631
	KNN	65.2	67.8	65.2	0.644
	Bayes	69.6	69.6	69.6	0.694
	LS-SVM	62.2	53.3	100.0	0.696
250	AdaBoost	69.6	71.3	69.6	0.692
	KNN	56.5	58.1	56.5	0.555
	Bayes	69.6	69.8	0.7	0.696
	LS-SVM	62.2	53.3	100.0	0.696
275	**AdaBoost**	**78.3**	**80.5**	**78.3**	**0.780**
	KNN	60.9	62.1	60.9	0.604
	Bayes	69.6	69.8	69.6	0.696
	LS-SVM	62.2	53.3	100.0	0.696
300	AdaBoost	69.6	71.3	69.6	0.692
	KNN	65.2	67.8	65.2	0.644
	Bayes	69.6	69.8	69.6	0.696
	LS-SVM	62.2	54.2	81.3	0.65
1556	AdaBoost	75.0	68.8	91.7	0.786
	KNN	60.9	56.3	81.8	0.667
	Bayes	73.9	72.7	72.7	0.727
	LS-SVM	56.8	50.0	93.8	0.652

*Bold font represents the best model*.

From the perspective of the number of characteristic wavelengths, when the number is 10, the best performer is the KNN classifier, with accuracy, precision, recall, and *F*_1_-score 69.6%, 69.8%, 69.6%, and 0.696. When the number is 50, LS-SVM performs best according to the accuracy of 67.6%, precision of 57.1%, recall of 100%, and *F*_1_-score 0.727. When the number is 100, Adaboost performs best while the best performer belongs to Bayes when the number is 200. As for the number 250, the results of AdaBoost are as good as Bayes. Finally, AdaBoost still stands out among four classifiers when under the condition of 300 characteristic wavelengths. From Table 1, it can be found that when the number of characteristic wavelengths is either too small or too large, the performance of every different classifier is not as good as the situation when the number is proper, from the perspective of four classifiers.

Compared to the results of original wavelengths number 1,556 without any procession, the best results of AdaBoost, KNN, and LS-SVM happen when they are through characteristic wavelengths selection, however, except Bayes. But after weighing the wavelength reduction and performance, it is reasonable to think that characteristic wavelength selection also works for Bayes.

**Figure 5:**
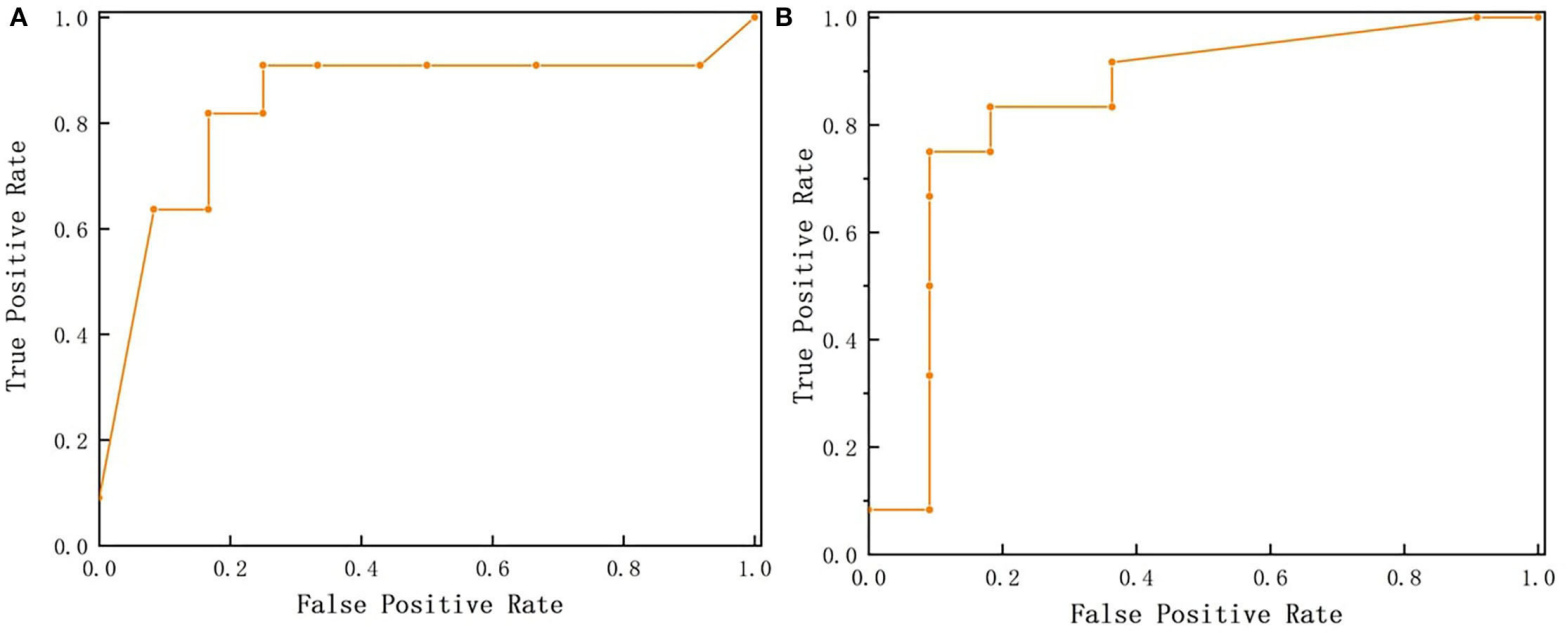
Receiver Operating Characteristic (ROC) curves. **(A)** is ROC curve of positive samples (SSC beyond 9 Brix) while **(B)** is ROC curve of negative samples (SSC below 9 Brix).

The ROC of positive and negative samples of the test set is shown in Figure 5. It can be seen that the ROC curves of positive and negative samples are all close to the upper left corner, and the total AUC is 0.841, indicating that the model has good robustness and can adapt flexibly to the uneven distribution of positive and negative samples in actual situations.

Too many spectral features bring information redundancy, and too few spectral features bring information loss. Based on the experimental results, for this classification task, the optimal number of spectral features is 275. Compared to the other modeling methods, the AdaBoost method achieves the best performance at 275 wavelength numbers. This is because AdaBoost combines multiple weak classifiers in a reasonable way to make one strong classifier. The other three methods used in this paper just give one separate model.

## 4. Conclusion and Reflection

Based on NIR spectroscopy, the random frog algorithm, and AdaBoost algorithm, and taking citrus in Shanghai Chongming Island as the research object, this study focuses on the problems of binary classification between NIR spectra and Miyagawa Satsuma SSC. Nine Brix is selected as the threshold of being sweet or not and the samples are divided into the training set and test set. After selecting characteristic wavelengths through the RF algorithm, they are used to establish binary classification models by AdaBoost, LS-SVM, and other classifiers. According to their performance, the AdaBoost classifier is the optimum model, with accuracy, precision, recall, and *F*_1_-score 78.3%, 80.5%, 78.3%, and 0.780, respectively.

Analyzing the model performance, we find that the constructed model does not have a very high performance. Combined with the sampling process and the test results, two reasons may be summarized (1) due to the limited penetration depth of NIR and the thick skin of the fruit, most of the NIR light does not penetrate the skin to reach the fruit part; and (2) there are environment disturbances during sampling and instrument errors in the process of collecting spectra.

The constructed model has the potential to be embedded in portable NIR acquisition devices in the future, which can facilitate fruit farmers to judge the quality of the citrus and be conducive to improving the sale pricing system of citrus in Chongming Island, so as to maximize the sale profit of fruit-sellers.

## Data Availability Statement

The raw data supporting the conclusions of this article will be made available by the authors, without undue reservation.

## Author Contributions

CZ and MH: funding acquisition. YC, MH, WS, SJ, LW, BD, ZC, and CZ: methodology and validation. YC, WS, and MH: writing—original draft. YC, SJ, LW, MH, and CZ: writing—review and editing. FJ: providing citrus materials. All authors contributed to the article and approved the submitted version.

## Funding

This study is sponsored by Shanghai Agriculture Applied Technology Development Program (Grant No. X20200102), Shanghai Agricultural System Standard Development Program (2018-013), Agriculture Research System of Shanghai (Grant No. 201407), Shanghai Agriculture Applied Technology Development Program (Grant No. T20220103), the Science and Technology Commission of Shanghai Municipality (No. 19511120100), the GHfund B (No. 20210702), and the Fundamental Research Funds for the Central Universities.

## Conflict of Interest

The authors declare that the research was conducted in the absence of any commercial or financial relationships that could be construed as a potential conflict of interest.

## Publisher's Note

All claims expressed in this article are solely those of the authors and do not necessarily represent those of their affiliated organizations, or those of the publisher, the editors and the reviewers. Any product that may be evaluated in this article, or claim that may be made by its manufacturer, is not guaranteed or endorsed by the publisher.
